# Low-density lipoprotein cholesterol-to-lymphocyte count ratio (LLR) is a promising novel predictor of postoperative new-onset deep vein thrombosis following open wedge high tibial osteotomy: a propensity score-matched analysis

**DOI:** 10.1186/s12959-024-00635-2

**Published:** 2024-07-16

**Authors:** Haichuan Guo, Chengsi Li, Hao Wu, Meixin Ma, Ruoxuan Zhu, Maolin Wang, Bin Yang, Naihao Pan, Yanbin Zhu, Juan Wang

**Affiliations:** 1 Department of Orthopedic Surgery, the 3rd Hospital of Hebei Medical University, NO.139 Ziqiang Road, Shijiazhuang, Hebei 050051 People’s Republic of China; 2 Department of Information Engineering, Shijiazhuang College of Applied Technology, Hebei, 050086 People’s Republic of China; 3 College of Letters & Science, University of California, Berkeley, Berkeley, CA 94720 USA; 4 Orthopedic Research Institute of Hebei Province, Key Laboratory of Biomechanics of Hebei Province, Shijiazhuang, 050051 Hebei People’s Republic of China

**Keywords:** Open wedge high tibial osteotomy, DVT, LDL-C, LYM, Novel predictor

## Abstract

**Background:**

The association of low-density lipoprotein cholesterol (LDL-C) and lymphocyte counts with the development of deep vein thrombosis (DVT) has been demonstrated in many fields but remains lacking in open wedge high tibial osteotomy (OWHTO). This study aimed to assess the predictive value of LDL-C to lymphocyte count ratio (LLR) in screening for postoperative new-onset DVT.

**Methods:**

Clinical data were retrospectively collected from patients who underwent OWHTO between June 2018 and May 2023. The limited restricted cubic spline (RCS) was conducted to evaluate the nonlinear relationship between LLR and the risk of postoperative new-onset DVT. The receiver operating characteristic (ROC) curves were plotted and the predictive value of biomarkers was assessed. After adjusting for intergroup confounders by propensity score matching, the univariate logistic regression was applied to assess the association between LLR and DVT.

**Results:**

1293 eligible patients were included. RCS analysis showed a linear positive correlation between LLR and the risk of DVT (*P* for overall = 0.008). We identified LLR had an area under the curve of 0.607, accuracy of 74.3%, sensitivity of 38.5%, and specificity of 80.7%, and LLR > 1.75 was independently associated with a 1.45-fold risk of DVT (95% CI: 1.01–2.08, *P* = 0.045). Furthermore, significant heterogeneities were observed in the subgroups of age, BMI, diabetes mellitus, hypertension, Kellgren-Lawrence grade, the American Society of Anesthesiologists (ASA) score, and intraoperative osteotomy correction size.

**Conclusion:**

LLR is a valuable biomarker for predicting postoperative new-onset DVT in patients with OWHTO, and routine screening is expected to yield positive benefits.

## Introduction

Postoperative new-onset deep vein thrombosis (DVT) is a highly prevalent (2.4-44.7%) and fatal complication following medial open wedge high tibial osteotomy (OWHTO) [1–4]. If not diagnosed and treated promptly, DVT can progress to post-thrombotic syndrome, which can lead to pulmonary embolism (PE) or even death [5]. More than 75% of DVT events are reported to be asymptomatic [6]. Furthermore, the OWHTO surgical procedure is performed on the medial side of the proximal tibia, thus making it difficult to differentiate between DVT symptoms and postoperative swelling and pain at the surgical site, and early diagnosis and timely targeted intervention are not easily achieved [7]. To avoid adverse prognostic events and the risk of delayed discharge, it makes improving the ability to diagnose or predict postoperative DVT following OWHTO is an ongoing goal for orthopedic surgeons.

Predictive modeling using baseline patient characteristics and hematological biomarkers, alone or in combination, is a cost-effective strategy to address this challenge non-invasively [8–10]. The excellent sensitivity of the D-dimer has made it the most commonly used parameter to screen for DVT in clinical practice [11]. Nevertheless, its low specificity severely hampers its further application in the diagnosis of DVT due to the simultaneous influence of organic trauma and inflammatory reaction [12]. Hence, exploring biomarkers with a higher diagnostic efficacy is currently a hot topic in DVT research [13–15]. A growing body of literature reveals the potential of low-density lipoprotein cholesterol (LDL-C) and lymphocyte count (LYM) in predicting thrombosis events [16–18]. For example, in patients with early-stage breast cancer, high LDL-C (> 3.6 mmol/L) increased the risk of catheter-related venous thrombosis by 2.36-fold [19]. On the other hand, lymphocytes play an important role in immunity and oxidative stress, and Ibebuogu et al. have found a significant correlation between decreased lymphocyte levels and intracardiac thrombosis in atrial fibrillation [20]. However, it is inappropriate to extrapolate the above findings in patients with malignancy or cardiovascular disease to postoperative new-onset DVT in orthopedic or even OWHTO patients. To our knowledge, no studies have evaluated in detail the role of these two biomarkers in predicting postoperative new-onset DVT following OWHTO. In addition, no study has attempted to explore the predictive value of the LDL-C to LYM ratio (LLR) in DVT screening.

Given the above, the aim of this study was to assess the predictive value of LDL-C and LYM for postoperative new-onset DVT after OWHTO and to further explore whether LLR might be a promising novel predictor.

## Materials and methods

### Patients

This was a single-institution retrospective study with inclusion criteria of consecutive patients undergoing OWHTO stabilized with a medial locking plate system for the treatment of medial compartment knee osteoarthritis between September 2017 and March 2023 at the Third Hospital of Hebei Medical University. Exclusion criteria were (1) incomplete data, (2) previous history of DVT and/or PE, (3) DVT diagnosed by preoperative duplex ultrasonography (DUS) or absence of DUS, (4) anticoagulant or antiplatelet therapy within the 3 months prior to admission, and (5) comorbidities with malignant tumors, history of knee trauma, or surgery. This study was conducted in accordance with the principles of the Declaration of Helsinki, and all processes adhered to the Strengthening the Reporting of Cohort Studies in Surgery (STROCSS) guidelines. The institutional ethic committee of the Third Hospital of Hebei Medical University accepted the study. All participants provided written informed consent and were anonymized for analysis.

### Surgical procedures

General laryngeal mask anesthesia combined with femoral nerve block was used for anesthesia, and the body position was supine. A balloon tourniquet was applied at the root of the thigh during the procedure, and the air pressure was set to 280 mmHg. Arthroscopy was performed routinely and intra-articular management was performed when necessary. Then, a longitudinal incision was made medial to the proximal tibia, the pes anserine tendon was preserved during exposure, and the superficial layer of the medial collateral ligament was released. The horizontal osteotomy plane was established above the pes anserine tendon insertion and parallel to the retroversion direction of the tibial plateau. The upper osteotomy was at a 110^°^ Angle to the horizontal osteotomy plane. After the biplanar osteotomy was completed, the osteotomy gap was slowly opened. According to the degeneration of the medial septal articular cartilage, the target hip-knee-ankle angle (HKA) was determined to be 180^°^-182^°^ and fixed with a T-locking compression plate and screws. The drainage tube was placed within 24 h after the operation, and partial weight bearing could be performed after extubation.

### Diagnosis and management of postoperative new-onset DVT

The diagnosis of postoperative new-onset DVT was made and verified by sonographers with at least 5 years of experience using the same set of equipment and, in the event of disagreement, by a senior sonographer. The criteria for the diagnosis of DVT by DUS were direct visualization of an intraventricular thrombus, venous incompressibility, venous dilatation greater than the diameter of the adjacent artery, reduced or absent enhancement of blood flow, and absence of spontaneous blood flow [21]. In accordance with departmental policy, the affected limbs were elevated approximately 30 cm after surgery, and a prophylactic subcutaneous injection of enoxaparin sodium at a dose of 4,000 AxaIU once daily was started within 12 h. Intermittent pneumatic compression therapy of both lower limbs was started within 24 h after the operation to improve blood circulation. Venous blood was sampled early in the morning of the second postoperative day for hematological parameters and to determine LLR levels. DUS examination was typically performed on the third postoperative day to identify the development of DVT. Prophylactic anticoagulation was continued in DVT-negative patients, and a therapeutic dose of anticoagulation was doubled in patients with a positive result (enoxaparin sodium injection at 4000 AxaIU twice daily). In addition, patients were instructed to perform ankle pumps and straight leg-raising exercises to prevent the development of DVT.

### Data collection

Clinical data were collected in five fields: demographics, medical comorbidities, preoperative and postoperative radiographs, surgical-related variables, and laboratory biomarkers. Demographics included gender, age, and calculated body mass index (BMI). Data on medical comorbidities encompassed hypertension, diabetes mellitus, heart disease, Hepatopathy, Cerebrovascular disease, Nephropathy, Operation history, Allergic history, Current smoking, and Alcohol consumption. The severity of knee osteoarthritis was evaluated radiologically using the Kellgren-Lawrence (K-L) grading system [22]. Surgical-related variables included the American Society of Anesthesiologists (ASA) score, operative duration, correction size, tourniquet time, and intraoperative blood loss. Laboratory biomarkers included hemoglobin concentration (HGB), platelet count (PLT), lymphocyte count (LYM), albumin (ALB), serum sodium concentration (Na^+^), fasting blood glucose (FBG), low-density lipoprotein cholesterol (LDL-C), LDL-C to LYM ratio (LLR), prothrombin time (PT), activated partial thromboplastin time (APTT), antithrombin III (AT III), fibrinogen (FIB), D-dimer level, and hypersensitive C-reactive protein (HCRP). The Youden’s index was applied to determine the optimal cut-off values for HCRP, LDL-C, LYM, LLR, and D-dimer. Each of these biomarkers was measured using the method recommended by the instrument manufacturer, with a complete blood count test using a hematology analyzer (UniCel DxH 800; Beckman Coulter, Brea, CA, USA), and coagulation using an ACL TOP 750 coagulometer (Instrument Laboratory, Bedford MA, USA) for coagulation studies and an AU5800 autoanalyzer (Beckman Coulter) for biochemical tests. If the patient underwent multiple hematology tests prior to the postoperative DUS examination, the closest was collected in this study.

### Statistical analysis

The Kolmogorov-Smirnov test was used to evaluate the normality of continuous variables. Normal distribution data were expressed as mean ± standard deviation (SD) and further analyzed by Student’s t-test. Skewed distribution data were presented as the median and interquartile range (IQR) and further analyzed by means of the Mann-Whitney test. Categorical variables were presented as numbers and percentages (%) and statistically analyzed using the chi-squared test or Fisher’s exact test. Postoperative new-onset DVT was the outcome event, and restricted cubic splines with 4 knots were used to evaluate the possible linear or non-linear relations. The receiver operating characteristic (ROC) curve was used to assess the diagnostic efficacy of LDL-C, LYM, LLR, and D-dimer. Diagnostic ability was quantified by the area under the ROC curve (AUC) [23]. The DeLong statistical test was performed using the roc.test function in the pROC package to compare the differences between AUCs [24]. The optimal cut-off values of each index were determined by calculating the Youden index, and the accuracy, sensitivity, specificity, positive predictive value (PPV), negative predictive value (NPV), and the corresponding 95% confidence interval (95%CI) were evaluated and compared using McNemar’s test [25, 26].

According to the optimal cut-off value of LLR (1.75), all patients were divided into “low LLR” and “high LLR” groups. Propensity score matching (PSM) was used to eliminate the interference of other confounding factors between the two groups as much as possible [27]. Propensity scores were calculated for all patients using a multiple logistic regression model with a matching pattern of 1:1 nearest neighbor matching algorithm and caliper width of 0.02. The standardized mean difference (SMD) was calculated and visualized to illustrate the covariate balance before and after PSM, and SMD > 0.1 indicated an imbalance between groups. Univariate logistic regression analysis was used to evaluate the association between LLR and DVT, and the Odds ratio (OR) value and 95% CI were obtained.

Subgroup analyses were performed to further evaluate the differences in the diagnostic value of LLR in different populations. In this study, the post-PSM cohort was divided into several subgroups based on age, BMI, diabetes, hypertension, K-L grade, ASA score, and osteotomy size. Univariate Logistic regression analyses were performed in each subgroup and the interaction between LLR level and each grouped covariate was assessed. All analyses were performed by SPSS version 26.0 (IBM Corp, Armonk, NY, USA) and R software version 4.2.1 (R Foundation for Statistical Computing, Vienna, Austria). A two-tailed *P* value of less than 0.05 was considered to indicate a level of statistical significance.

## Results

**Fig. 1:**
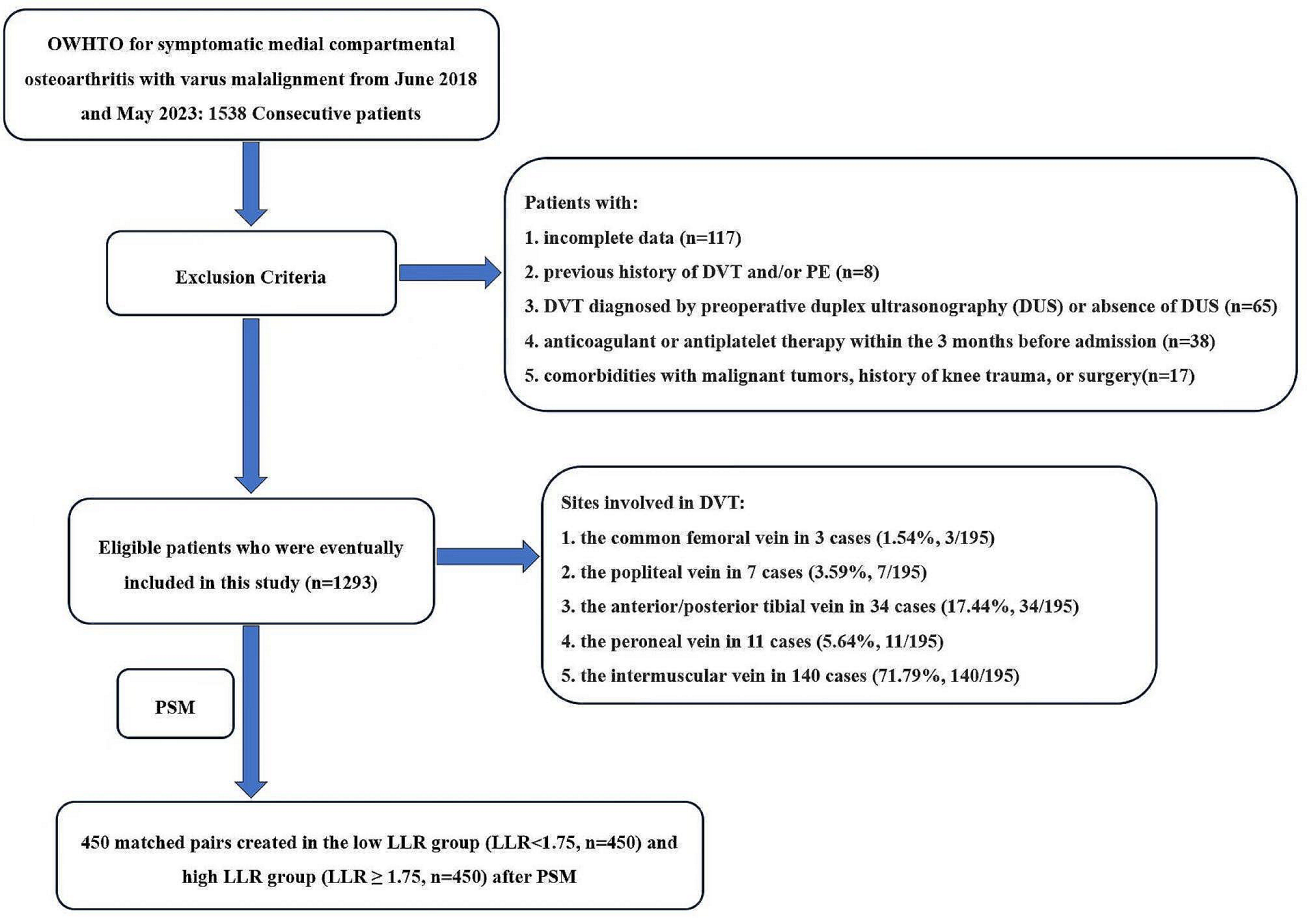
Flowchart of patient selection for this study

Figure 1 illustrates the flow chart of the study, with a total of 1538 OWTHO patients initially selected. According to the exclusion criteria, 1293 patients were finally retained for analysis. After the DUS examination, 195 patients (15.1%, 195/1293) were diagnosed with postoperative new-onset DVT. DVT involved the common femoral vein in 3 cases (1.54%, 3/195), the popliteal vein in 7 cases (3.59%, 7/195), the anterior/posterior tibial vein in 34 cases (17.44%, 34/195), the peroneal vein in 11 cases (5.64%, 11/195), and the intermuscular vein in 140 cases (71.79%, 140/195). All patients with DVT were asymptomatic.

**Fig. 2:**
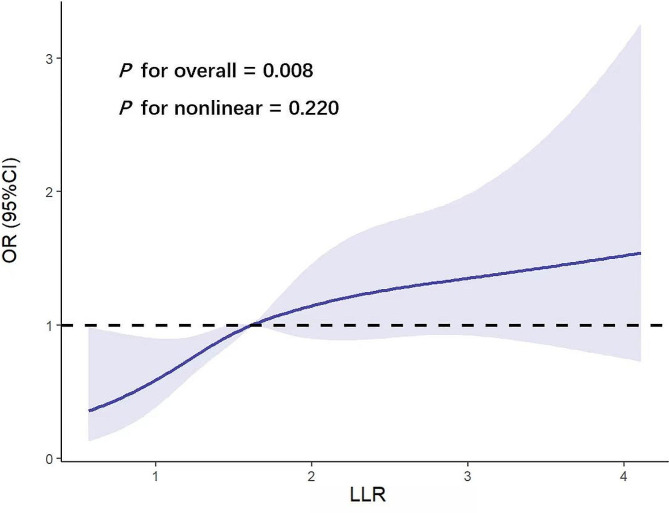
Associations of the LLR and the risk of postoperative new-onset DVT following OWHTO using restricted cubic spline

**Fig. 3:**
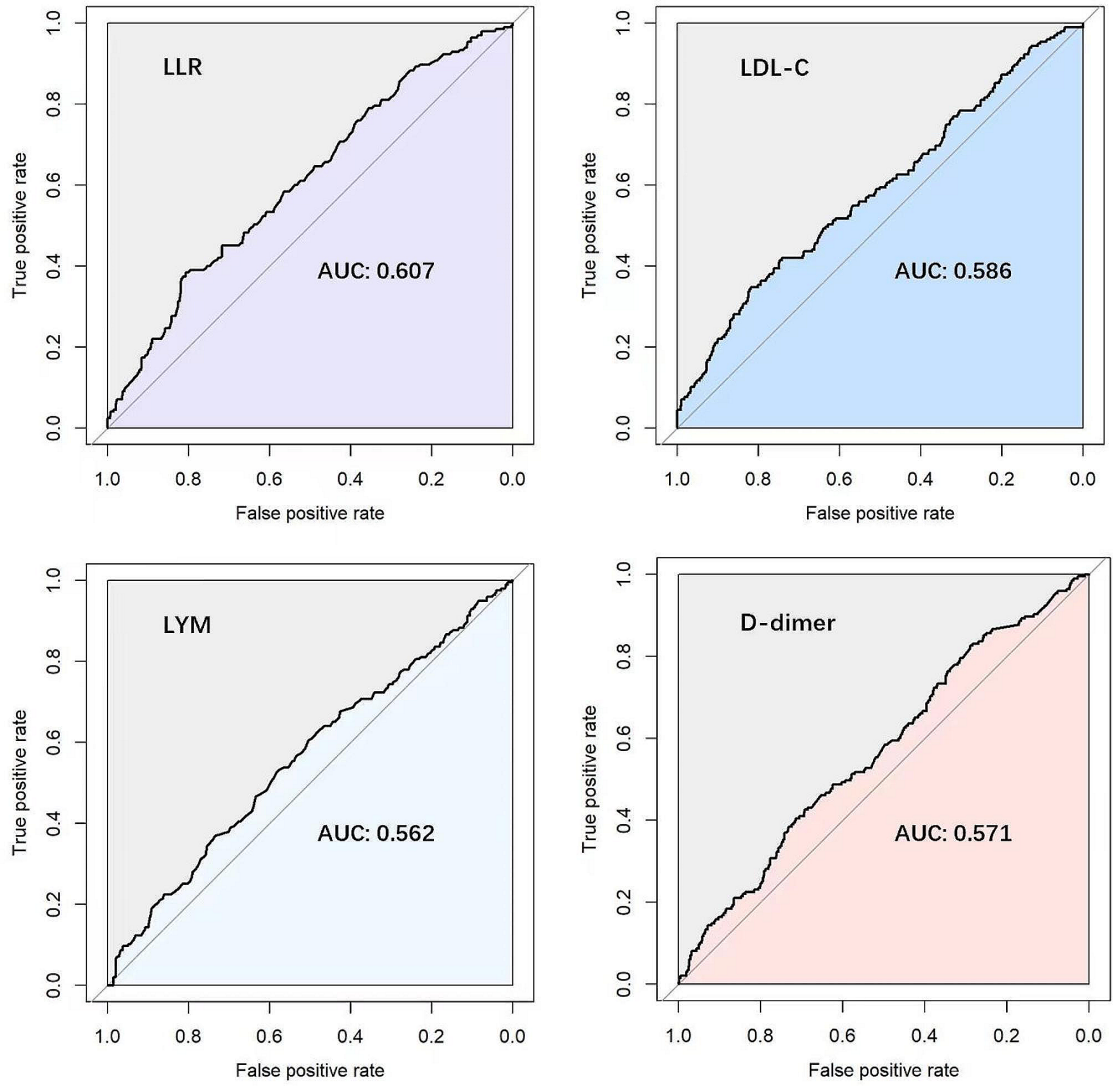
ROC curves for comparisons LLR, LDL-C, LYM, and D-dimer in OWHTO patients

RCS analysis (Fig. 2) was performed to visualize the dose-effect relationship between LLR and DVT risk. The result revealed a linear positive correlation between LLR and the risk of DVT (*P* for overall = 0.008). ROC curves (Fig. 3) were used to assess the diagnostic value of LDL-C, LYM, LLR, and D-dimer, and the area under the curve (AUC) was positively correlated with the diagnostic efficacy of each marker. Table 1 shows the cut-off values, accuracy, AUC, sensitivity, specificity, PPV, NPV, and corresponding 95% CI for the four predictors. we found that LLR possessed the highest AUC (0.607), LDL-C had the highest specificity (81.8%) and accuracy (74.7%), and LYM had the highest sensitivity (52.8%). We noted that LLR and LDL-C were characterized by high specificity and low sensitivity. Compared with LDL-C, LLR had slightly higher sensitivity (%, 38.5 vs. 34.9, *P* = 0.326) but slightly lower specificity (%, 80.7 vs. 81.8, *P* = 0.686). The AUC of LLR was higher than that of LDL-C (0.608 vs. 0.587, *P* = 0.316), LYM (0.608 vs. 0.562, *P* = 0.013) and D -dimer (0.608 vs. 0.571, *P* = 0.193). In addition, LLR possessed higher specificity (%, 80.7 vs. 71.3, *P* = 0.029), accuracy (%, 74.3 vs. 66.7, *P* = 0.042), PPV (%, 26.1 vs. 20.1, *P* = 0.022), and NPV (%, 88.1 vs. 87.1, *P* = 0.755) compared with D-dimer.

**Table 1:** Evaluation of characteristic parameters in four biomarkers

Variables	Cut-off value	Accuracy	AUC (95% CI)	Sensitivity (%,95% CI)	Specificity (%,95% CI)	PPV (%,95% CI)	NPV (%,95% CI)
LLR	1.75	74.3%	**0.607** (0.566, 0.652)	38.5 (31.7, 45.7)	80.7 (78.2, 83.0)	26.1 (21.2, 31.7)	88.1 (85.9, 89.9)
LDL-C	3.10	74.7%	0.586 (0.542, 0.633)	34.9 (28.3, 42.1)	**81.8** (79.3, 84.0)	25.4 (20.4, 31.1)	87.6 (85.4, 89.5)
LYM	1.79	57.5%	0.562 (0.517, 0.607)	**52.8** (45.6, 60.0)	58.3 (55.3, 61.2)	18.4 (15.3, 21.9)	87.4 (84.8, 89.7)
D-dimer	1.38	66.7%	0.571 (0.527, 0.614)	40.5 (33.6, 47.8)	71.3 (68.5, 74.0)	20.1 (16.3, 24.4)	87.1 (84.7, 89.2)

**Abbreviations**: AUC, area under the curve; CI, confidence interval; PPV, positive predictive value; NPV, negative predictive value; LDL-C, low-density lipoprotein cholesterol; LYM, lymphocyte count; LLR, low-density lipoprotein cholesterol -to- lymphocyte count ratio

**Fig. 4:**
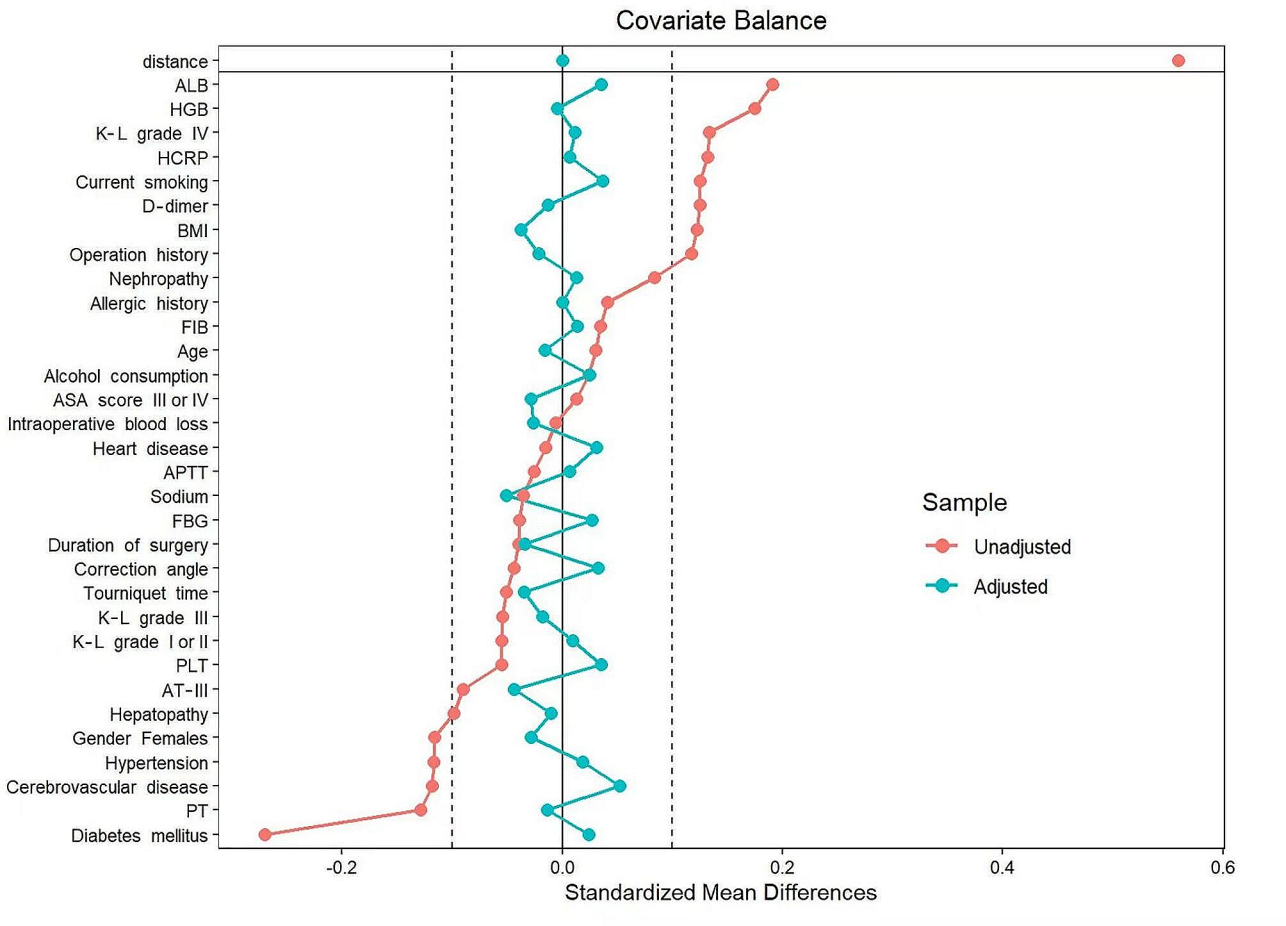
Standardized mean differences (SMD) between the groups of low LLR and high LLR across baseline clinical data. Absolute value of SMD < 0.1 indicated adequate between-group balance. ALB, albumin; HGB, hemoglobin, reference range: Females, 110–150 g/L; males, 120–160 g/L; K-L grade, Kellgren-Lawrence grade; HCRP, high sensitivity C-reactive protein; BMI, body mass index; FIB, fibrinogen; ASA, American Society of Anesthesiologists; APTT, activated partial thromboplastin time; FBG, fasting blood glucose; PLT, platelet; AT III, antithrombin III; PT, prothrombin time

The optimal threshold of LLR was 1.75, based on which patients were categorized into “low LLR” and “high LLR” groups. Table 2 presented the differences in baseline characteristics comparing the two groups before and after PSM. The results showed that 759 patients (58.7%) were included in the low LLR group and 534 patients (41.3%) in the high LLR group in the pre-PSM dataset. Univariate analysis revealed significant differences between groups for covariates such as gender, obesity, hypertension, diabetes, surgical history, smoking, K-L classification, HGB, ALB, PT, D-dimer, and HCRP. After PSM, 900 cases were retained with a group match of 1:1. Univariate analysis showed that none of the covariates were statistically significant between the two groups. In addition, Fig. 4 visualized the SMD distributions of all covariates before and after PSM, with SMD absolute values < 0.1 indicating adequate between-group balance. It can be intuitively observed that all covariates in the post-PSM cohort are well-balanced. Univariate logistic regression revealed that high LLR was significantly associated with a 1.45-fold increase in the risk of DVT (OR: 1.45, 95% CI: 1.01–2.08, *P* = 0.045).

**Table 2:** Comparison of the baseline characteristics of patients before and after PSM

Variables	Before PSM		SMD*	*P* value	After PSM		SMD*	*P* value
	Low LLR (*n* = 759)	High LLR (*n* = 534)			Low LLR (*n* = 450)	High LLR (*n* = 450)		
Gender (female)	542 (71.4%)	352 (65.9%)	0.119	0.041	311 (69.1%)	304 (67.6%)	0.033	0.667
Age (years)	58.5 ± 8.2	58.7 ± 7.5	0.029	0.611	58.5 ± 8.3	58.6 ± 7.4	0.012	0.859
BMI (kg/m^2^) > 28	223 (29.4%)	188 (35.2%)	0.125	0.031	152 (33.8%)	135 (30.0%)	0.081	0.252
Hypertension	308 (40.6%)	187 (35.0%)	0.115	0.049	157 (34.9%)	166 (36.9%)	0.042	0.578
Diabetes mellitus	119 (15.7%)	44 (8.2%)	0.231	< 0.001	36 (8.0%)	43 (9.6%)	0.055	0.480
Heart disease	70 (9.2%)	47 (8.8%)	0.015	0.872	33 (7.3%)	38 (8.4%)	0.041	0.621
Hepatopathy	53 (7.0%)	26 (4.9%)	0.090	0.149	22 (4.9%)	25 (5.6%)	0.030	0.764
Cerebrovascular disease	38 (5.0%)	16 (3.0%)	0.103	0.101	11 (2.4%)	16 (3.6%)	0.065	0.434
Nephropathy	13 (1.7%)	17 (3.2%)	0.095	0.123	7 (1.6%)	9 (2.0%)	0.034	0.801
Operation history	136 (17.9%)	122 (22.8%)	0.123	0.035	105 (23.3%)	98 (21.8%)	0.037	0.632
Allergic history	29 (3.8%)	25 (4.7%)	0.043	0.535	19 (4.2%)	20 (4.4%)	0.011	1.000
Current smoking	86 (11.3%)	85 (15.9%)	0.134	0.021	56 (12.4%)	63 (14.0%)	0.046	0.555
Alcohol consumption	110 (14.5%)	82 (15.4%)	0.024	0.726	66 (14.7%)	68 (15.1%)	0.012	0.925
K-L grade			0.142	0.041			0.042	0.818
I-II	264 (34.8%)	172 (32.2%)			155 (34.4%)	153 (34.0%)		
III	386 (50.9%)	257 (48.1%)			225 (50.0%)	220 (48.9%)		
IV	109 (14.4%)	105 (19.7%)			70 (15.6%)	77 (17.1%)		
ASA score			0.013	0.880			0.006	1.000
I-II	622 (81.9%)	435 (81.5%)			370 (82.2%)	371 (82.4%)		
III-IV	137 (18.1%)	99 (18.5%)			80 (17.8%)	79 (17.6%)		
Operative duration (min)	97.8 ± 38.3	99.3 ± 48.0	0.035	0.543	99.8 ± 52.7	97.3 ± 36.3	0.055	0.406
Correction size	9.9 ± 2.6	10.1 ± 2.6	0.044	0.440	9.9 ± 2.6	10.0 ± 2.6	0.029	0.660
Tourniquet time (min)	84.4 ± 34.3	86.1 ± 44.7	0.043	0.452	86.3 ± 49.8	83.7 ± 32.2	0.062	0.353
Intraoperative blood loss (ml)	112.9 ± 64.1	112.5 ± 62.2	0.006	0.920	113.9 ± 63.5	111.5 ± 60.8	0.039	0.557
HGB (< lower limit)	336 (44.3%)	283 (53.0%)	0.175	0.002	210 (46.7%)	218 (48.4%)	0.036	0.640
PLT (> 300 × 10^9^/L)	97 (12.8%)	59 (11.0%)	0.053	0.393	45 (10.0%)	53 (11.8%)	0.057	0.454
ALB (< 35 g/L)	320 (42.2%)	276 (51.7%)	0.192	0.001	202 (44.9%)	211 (46.9%)	0.040	0.593
Sodium (< 135mmol/L)	64 (8.4%)	40 (7.5%)	0.035	0.611	38 (8.4%)	33 (7.3%)	0.041	0.621
FBG (> 6.1mmol/L)	177 (23.3%)	116 (21.7%)	0.038	0.543	86 (19.1%)	101 (22.4%)	0.082	0.250
PT (> 12.5s)	126 (16.6%)	66 (12.4%)	0.121	0.042	62 (13.8%)	62 (13.8%)	< 0.001	1.000
APTT (< 28s)	106 (14.0%)	70 (13.1%)	0.025	0.719	60 (13.3%)	61 (13.6%)	0.007	1.000
AT III (< 80%)	70 (9.2%)	37 (6.9%)	0.084	0.170	36 (8.0%)	32 (7.1%)	0.034	0.705
FIB (> 4.4 g/L)	17 (2.2%)	15 (2.8%)	0.036	0.641	10 (2.2%)	11 (2.4%)	0.015	1.000
D-dimer (> 1.38 mg/L)	345 (45.5%)	276 (51.7%)	0.125	0.031	221 (49.1%)	223 (49.6%)	0.009	0.947
HCRP (> 57.7 mg/L)	73 (9.6%)	76 (14.2%)	0.143	0.014	52 (11.6%)	54 (12.0%)	0.014	0.918

**Note**: *Absolute SMD > 0.1 indicated favorable intergroup balance

**Abbreviations**: BMI, body mass index; K-L grade, Kellgren-Lawrence grade; ASA, American Society of Anesthesiologists; HGB, hemoglobin, reference range: Females, 110–150 g/L; males, 120–160 g/L; PLT, platelet; ALB, albumin; FBG, fasting blood glucose; PT, prothrombin time; APTT, activated partial thromboplastin time; AT III, antithrombin III; FIB, fibrinogen; HCRP, high sensitivity C-reactive protein

**Fig. 5:**
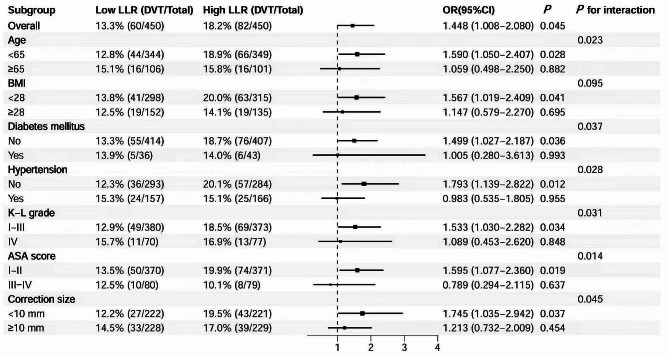
The forest plot for subgroup analysis represents the OR and 95% confidence interval (CI) of DVT associated with high LLR. DVT, deep vein thrombosis; OR (Odds ratio); CI (confidence interval); K-L grade, Kellgren-Lawrence grade; ASA, American Society of Anesthesiologists

Finally, forest plots (Fig. 5) were developed to present the detailed results of the subgroup analyses, identifying significantly heterogeneous populations. The high LLR was positively associated with the risk of development of postoperative new-onset DVT across all subgroups (all OR > 1). Compared with other groups, low age (1.59 vs. 1.06), non-obesity (1.02 vs. 2.41), non-diabetes mellitus (1.50 vs. 1.01), non-hypertension (1.79 vs. 0.98), low Kellgren -Lawrence (K-L) grade (1.53 vs. 1.09), the American society of anesthesiologists (ASA) score I-II (1.60 vs. 0.79), and intraoperative osteotomy correction size < 10 mm (1.75 vs. 1.21) had a higher relative risk of postoperative new-onset DVT associated with high LLR (*P* for interaction < 0.05).

In summary, LLR was positively associated with the risk of new DVT after OWHTO. LLR > 1.75 possessed excellent diagnostic efficacy and increased the risk of DVT by 1.45-fold after adjustment for confounders. Furthermore, this risk estimate was strengthened in specific subgroups.

## Discussion

To our knowledge, this is the first study that found the linear positive correlation between LLR and the risk of postoperative new-onset DVT in patients with OWHTO and to evaluate the diagnostic ability of LDL-C, LYM, and LLR. In comparing the predictive performance of LDL-C, LYM, LLR, and D-dimer, we identified that LDL-C possessed the highest specificity, and LYM the highest sensitivity. LLR has complementary advantages with both of these biomarkers. Compared with D-dimer, LLR, a novel biomarker, has a higher AUC and specificity in predicting postoperative new-onset DVT. We found that high LLR (> 1.75) was independently associated with a 1.45-fold risk of developing DVT in the post-PSM cohort. Compared with controls, the risk of developing DVT associated with high LLR was higher in the subgroups of age < 65 years, non-obesity, non-diabetes mellitus, non-hypertension, K-L grades I-III, ASA scores I-II, and corrected size < 10 mm.

High LDL-C is a common feature of dyslipidemia. In the field of trauma and oncology, LDL-C has been established as an independent predictor of DVT [19, 28], and the present study further revealed a potential association between high levels of LDL-C and an increased risk of postoperative new-onset DVT following OWHTO. This is related to the fact that LDL-C particles can cause chronic low-grade inflammation, platelet-activated aggregation, as well as decreased nitric oxide bioavailability and thus induced endothelial dysfunction [29–31]. On the other hand, reduced lymphocyte counts are an independent risk factor for poor prognosis in many diseases [32–35]. A prospective study by Ibebuogu et al. found that peripheral blood lymphocyte levels were significantly lower in patients with D-dimer-positive thrombosis compared to those without thrombosis [20], suggesting a potential negative correlation between lymphocyte count levels and thrombosis. Firstly, reduced peripheral blood lymphocyte levels can cause immunosuppression and impaired regulation of the inflammatory response, leading to excessive or insufficient inflammatory response and promoting thrombosis [36]. In addition, certain lymphocyte subsets, such as regulatory T cells (Treg cells) and natural killer T cells (NKT cells), can be directly involved in the regulation of the coagulation system and inhibit clotting factor activity [37, 38]. Therefore, decreased lymphocyte counts can reduce anticoagulant capacity and increase the risk of thrombosis.

In recent years, LDL-C and LYM in combination with other biomarkers have been a hot topic for clinical disease risk factor analysis. For example, for LDL-C, the LDL-C to apolipoprotein B (apo B) ratio had been demonstrated to be independently associated with all-cause mortality and cardiovascular events in peritoneal dialysis patients [39], and The High-density lipoprotein cholesterol (HDL-C) to LDL-C ratio can be applied to early assessment of disease severity and outcome in patients with acute pancreatitis admitted to ICU [40]. As for LYM, the Platelet Count to LYM Ratio has excellent diagnostic power in predicting periprosthetic joint infection in patients undergoing total knee arthroplasty (TKA) [41], while the fibrinogen to LYM ratio has been established as a new prognostic indicator for patients after radical hepatocellular carcinoma operation [42]. Different from previous studies, this study is the first attempt to use the LDL-C to LYM ratio, which adequately demonstrates the good thrombus prediction value of the LDL-C to LYM ratio in the field of osteotomies and orthopedics.

The different distributions of OWHTO patient characteristics increase heterogeneity and may lead to differences in the predictive efficacy of the LLR for DVT in different population groups. After eliminating confounding effects, subgroup analysis revealed that the predictive value of LLR was valid in most subgroups, especially in those with younger age, better physical condition, and smaller intraoperative osteotomy correction size. The underlying mechanism may be that high LLR contributes to the development of DVT in combination with pathologic conditions or complex surgical procedures such as advanced age, poor physical condition, and large osteotomy size. The correlation between high LLR and DVT was highlighted after excluding the effects of these pathologic conditions. Therefore, it is reasonable to conclude that LLR is more effective in predicting postoperative new-onset DVT in middle-aged OWHTO patients with relatively better physical conditions and smaller osteotomy sizes and warrants further investigation.

It is worth noting that despite its favorable specificity for the diagnosis of postoperative new-onset DVT following OWHTO, the relatively low sensitivity and positive predictive value of the LLR suggest that the LLR is more suitable as an exclusionary indicator. Particularly for those sensitive populations in the subgroup analysis, special attention and focused high-risk screening are recommended for implementation. The classical D-dimer and Caprini scores can be combined simultaneously to improve the screening rate for DVT. If necessary, targeted and focused prophylaxis based on routine anticoagulation can be considered.

This study’s strengths include identifying LLR as a highly specific novel predictor of postoperative new-onset DVT following OWHTO and confirming and validating their true association and degree of association using a large sample. However, several limitations of the study should be mentioned. Firstly, the present study did not take the use of lipid-lowering drugs into consideration. The retrospective study failed to analyze this factor because the details of the type, dosage, and treatment course of lipid-lowering drugs were not well documented in the medical records. This may have affected the accuracy of the results to some extent, although the LDL-C-to-lymphocyte count ratio (LLR) itself showed excellent predictive value. Secondly, although 30 important parameters have been adjusted in this study, the potential effects of some unincluded variables may still leave residual confounding remaining, such as patients’ duration of absolute bed rest after surgery. Thirdly, the selection and recall biases were unavoidable because the accuracy of some variables depended on the patient’s perception of his or her own condition, such as allergy history. Fourthly, no further study was conducted to examine the potential impact of LLR on the occurrence of DVT extension or recanalization, which could offer valuable guidance in assessing and improving the prognosis of patients. Fifthly, the statistical analysis of this study was based on single-center data, which needs to be further validated and optimized in multicenter prospective studies with large samples.

## Conclusion

In this study, we identified LDL-C, LYM, and LLR as valuable biomarkers for predicting postoperative new-onset DVT in patients with OWHTO. In the post-PSM cohort, LLR, a novel high-specificity predictor (81.8%), was independently associated with a 1.45-fold risk of postoperative new-onset DVT following OWHTO and was more potent in a subgroup of patients who were younger, in better physical condition, and with smaller osteotomy size.

## Data Availability

All data for this study can be obtained from the corresponding authors on reasonable request.

## Abbreviations

LDL-C: Low-density lipoprotein cholesterol
LYM: Lymphocyte count
DVT: Deep vein thrombosis
OWHTO: Open wedge high tibial osteotomy
LLR: LDL-C to LYM ratio
RCS: Restricted cubic spline
ROC: Receiver operating characteristic
PSM: Propensity score matching
AUC: Area under the ROC curve
K-L: Kellgren-Lawrence
ASA: American Society of Anesthesiologists
PE: Pulmonary embolism
DUS: Duplex ultrasonography
STROCSS: Strengthening the Reporting of Cohort Studies in Surgery
BMI: Body mass index
HGB: Hemoglobin
PLT: Platelet
ALB: Albumin
FBG: Fasting blood glucose
PT: Prothrombin time
APTT: Activated partial thromboplastin time
AT III: Antithrombin III
FIB: Fibrinogen
HCRP: Hypersensitive C-reactive protein
SD: Standard deviation
IQR: Interquartile range
PPV: Positive predictive value
NPV: Negative predictive value
95% CI: 95% confidence interval
SMD: Standardized mean difference
OR: Odds ratio
